# Draft Genome Sequences of *Escherichia coli* Strains Isolated at Calving from the Uterus, Vagina, Vulva, and Rectoanal Junction of a Dairy Cow That Later Developed Metritis

**DOI:** 10.1128/genomeA.01511-16

**Published:** 2017-03-16

**Authors:** Soo Jin Jeon, Federico Cunha, Amber Ginn, KwangCheol Casey Jeong, Klibs N. Galvão

**Affiliations:** a Department of Large Animal Clinical Sciences, College of Veterinary Medicine, University of Florida, Gainesville, Florida, USA; b Department of Animal Sciences, University of Florida, Gainesville, Florida, USA; c D. H. Barron Reproductive and Perinatal Biology Research Program, University of Florida, Gainesville, Florida, USA

## Abstract

*Escherichia coli* is involved in the pathogenicity of metritis in cows. We report here the genome sequences of *E. coli* strains isolated at calving from the uterus, vagina, vulva, and rectoanal junction of a dairy cow that later developed metritis. The genomic similarities will give an insight into phylogenetic relationships among strains.

## GENOME ANNOUNCEMENT

Intrauterine* Escherichia coli* is a primary cause of uterine diseases, facilitating the growth of anaerobic pathogens in the uterus of dairy cows (1), and is known to invade the uterus through the type 1 pilus tip adhesion that is encoded in the *fimH* gene (2). Draft genome sequences of intrauterine* E. coli* strains associated with metritis have previously been reported (3, 4). Here, we document draft genome sequences of *E. coli* strains isolated from the uterus, vagina, vulva, and rectoanal junction of a dairy cow that later developed metritis.

Swabs from the uterus, vagina, vulva, and rectoanal junction were taken from a dairy cow (ID 8749) within 30 min after calving. The swabs were resuspended in 1 ml of Luria-Bertani broth (Sigma-Aldrich), and 200 µl of diluted sample was cultured overnight on CHROMagar *E. coli* (CHROMagar). The next day, colonies with blue color were PCR amplified for the *fimH* gene (forward primer, 5′-TGACCGTAAATGGTGGAGCC-3′; reverse primer, 5′-TGGCCTACAAAGGGCTAACG-3′). Five *fimH*-positive strains were randomly chosen in each body habitat and confirmed using Sanger sequencing. A *fimH* gene phylogenetic tree obtained from the neighbor-joining method indicated the presence of two groups of *E. coli* strains; however, only one group included strains found in all body habitats. Therefore, the group having *E. coli* strains from all body habitats was chosen for this study, and representative isolates from the uterus (KG-8), vagina (KG-10), vulva (KG-24), and rectoanal junction (KG-16) were selected for whole-genome sequencing.

Genomic DNA extraction was carried out using the DNeasy blood and tissue kit (Qiagen), according to the manufacturer’s instructions. Sequencing was performed using the Illumina MiSeq (Illumina, Inc.), with a 2 × 250-bp 500-cycle cartridge. There were 712,117 clusters and 1,424,234 reads in KG-8, with a genome coverage of 71×. There were 272,205 clusters and 544,410 reads in KG-10, with a genome coverage of 27×. There were 907,512 clusters and 1,815,024 reads in KG-24, with a genome coverage of 91×. There were 556,540 clusters and 1,113,080 reads in KG-16, with a genome coverage of 56×. After trimming of FastQ data using Sickle (5), with the length parameter set to 50 and quality set to 30, *de novo* assemblies were performed with SPAdes (6) using the k-mer values of 21, 33, 55, 77, 99, and 127. Assembled genomes were annotated using PATRIC (7). KG-8 consisted of 4,888,829 bp (50.72% G+C content), 4,885 protein-coding sequences (CDSs), 12 rRNA, and 80 tRNA, with 217 virulence factors. KG-10 consisted of 4,853,983 bp (50.87% G+C content), 4,854 CDSs, 11 rRNA, and 71 tRNA, with 220 virulence factors. KG-24 consisted of 4,879,321 bp (50.75% G+C content), 4,871 CDSs, 12 rRNA, and 79 tRNA, with 214 virulence factors. KG-16 consisted of 5,146,599 bp (50.59% G+C content), 5,194 CDSs, 10 rRNA, and 82 tRNA, with 219 virulence factors. All of these strains had a beta-lactamase gene which confers resistance to cephalosporins.

This is the first report of genome sequences of *E. coli* strains recovered from multiple body habitats at calving from a cow that later developed metritis. Comparative genome analysis among *E. coli* strains will help reveal the origin of intrauterine* E. coli*.

### Accession number(s).

The whole-genome sequences are available at DDBJ/EMBL/GenBank under the accession numbers MPAY00000000 (KG-8), MPDS00000000 (KG-10), MPBF00000000 (KG-24), and MPDR00000000 (KG-16).
